# Supplementary material for: Isogenic Human-Induced Pluripotent Stem-Cell-Derived Cardiomyocytes Reveal Activation of Wnt Signaling Pathways Underlying Intrinsic Cardiac Abnormalities in Rett Syndrome
Source: Int J Mol Sci. 2022 Dec 9;23(24):15609. doi: 10.3390/ijms232415609 (PMC9779632; doi:10.3390/ijms232415609)
Supplement: Supplementary file 1 [file ijms-23-15609-s001.zip › ijms-2013609-supplementary.pdf]

Table S1:

Pre-designed assay primers For Long QT syndrome specific genes

Target	Identifier (Catalog number)	Vender
**ANK1**	HS_ANK1_1_SG (QT00076958)	Qiagen
**AKAP9**	HS_AKAP9_1_SG (QT00090454)	Qiagen
**CACNA1C**	HS_CACNA1C_1_SG (QT00053480)	Qiagen
**CALM1**	HS_CALM1_1_SG (QT00997906)	Qiagen
**CALM2**	HS_CALM2_1_SG (QT00997913)	Qiagen
**CAV3**	HS_CAV3_1_SG (QT00204071)	Qiagen
**KCNE1**	HS_KCNE1_1_SG (QT01003226	Qiagen
**KCNE2**	HS_KCNE2_1_SG (QT00100044	Qiagen
**KCNH2**	HS_KCNH2_1_SG (QT01003254	Qiagen
**KCNJ2**	HS_KCNJ2_1_SG (QT00001022	Qiagen
**KCNJ5**	HS_KCNJ5_1_SG (QT00070406	Qiagen
**KCNQ1**	HS_KCNQ1_1_SG (QT00016065	Qiagen
**SCN4B**	HS_SCN4B_1_SG (QT00011802)	Qiagen
**SCN5A**	HS_SCN5A_1_SG (QT00091812	Qiagen
**SNTA1**	HS_SNTA1_1_SG (QT00008036)	Qiagen

Table S2: Oligonucleotides used in quantitative PCR analysis:

Target gene	Sequence (5'→3')
hTNNT2	Forward: GAATGAAGATCAGCTGAG<br>Reverse: TTATCGTTGATCCTGTTTC
WNT1	Forward: TACGCCAAAATCCGGGGATC<br>Reverse: ACGATCTTGCCGAAGAGGTG
WNT2	Forward: GCTACGACACCTCCCATGTC<br>Reverse: TGCACATCCAGAGCTTCCAG
WNT2B	Forward: GGCACGAGTGATCTGTGAC<br>Reverse: ATTGGTGCTGACACTCTCGG
WNT3A	Forward: GGAGATGGTGGTGGAGAAGC<br>Reverse: AGGCCTCGTAGTAGACCAGG
WNT4	Forward: AGGCCATCCTGACACACATG<br>Reverse: TTCTCCTTCAGTGCGTGACC
WNT5A	Forward: TAGCCTGAAGACATGCTGGC<br>Reverse: TGACCTGTACCAACTTGCCC
WNT6	Forward: GGAAGTGGTGGCAGAGCTAG<br>Reverse: TCTCCCGAATGTCCTGTTGC
WNT7B	Forward: AGAAGTCGCCCAACTACTGC<br>Reverse: TGGTGTACTGGTGGGTGTTG
WNT8A	Forward: AAGGCCAAGTATGACCAGGC<br>Reverse: CTCTGCGCTAGGAAGGAAGG
WNT8B	Forward: AAGGAGAAGTACCACGCAGC<br>Reverse: AGATGGAGCGAAAGGTGTCG
WNT9A	Forward: ACCGTGAGAAGAACTGCGAG<br>Reverse: CACTCCACATAGCAGCACC
WNT9B	Forward: TCAGGACCACGTGTAAGTGC<br>Reverse: ATAGCGCAGTTTCAGCACC
WNT10A	Forward: TTCTTCCTACTGCTGCTGGC<br>Reverse: TCAGGCCTGGCAATGTTAGG
NANOG	Forward: CAGAACTGTGTTCTCTTCCA<br>Reverse: GTTCAGGATGTTGGAGAGTT
OCT4	Forward: TCAGGAGATATGCAAAGCAG<br>Reverse: CACTGCAGGAACAAATTCTC
TBXT	Forward: CCTCGAATCCACATAGTGAG<br>Reverse: TCGTTCTGATAAGCAGTCAC
MESP1	Forward: AGTGAGCGGGAGAAACTG<br>Reverse: ACAGGTGGCCGATATAGC
ISL1	Forward: ACGTCTGATTTCCCTATGTG<br>Reverse: ATACTGATTACACTCCGCAC
NKX2.5	Forward: CTTCTATCCACGTGCCTAC<br>Reverse: TCTGTCTTCTCCAGCTCC
MEF2C	Forward: GCAACACCTACATAACATGC<br>Reverse: GACTTGATGTTGAGGCTTTG
GATA4	Forward: TTTGACGACTTCTCAGAAGG<br>Reverse: CGTTCATCTTGTGGTAGAGG
MYH6	Forward: AGGCCAAAGTAAAGGAGATG<br>Reverse: AATGTCCTTCTTGAGCTCTG
MYH7	Forward: GGAAGCAGAAGTATGAGGAG<br>Reverse: TTGAAGGTCTCCAGATGTTC
GAPDH	Forward: GTCTCCTCTGACTTCAACAGCG<br>Reverse: ACCACCCTGTTGCTGTAGCCAA

Figure S1, related to Figure 1

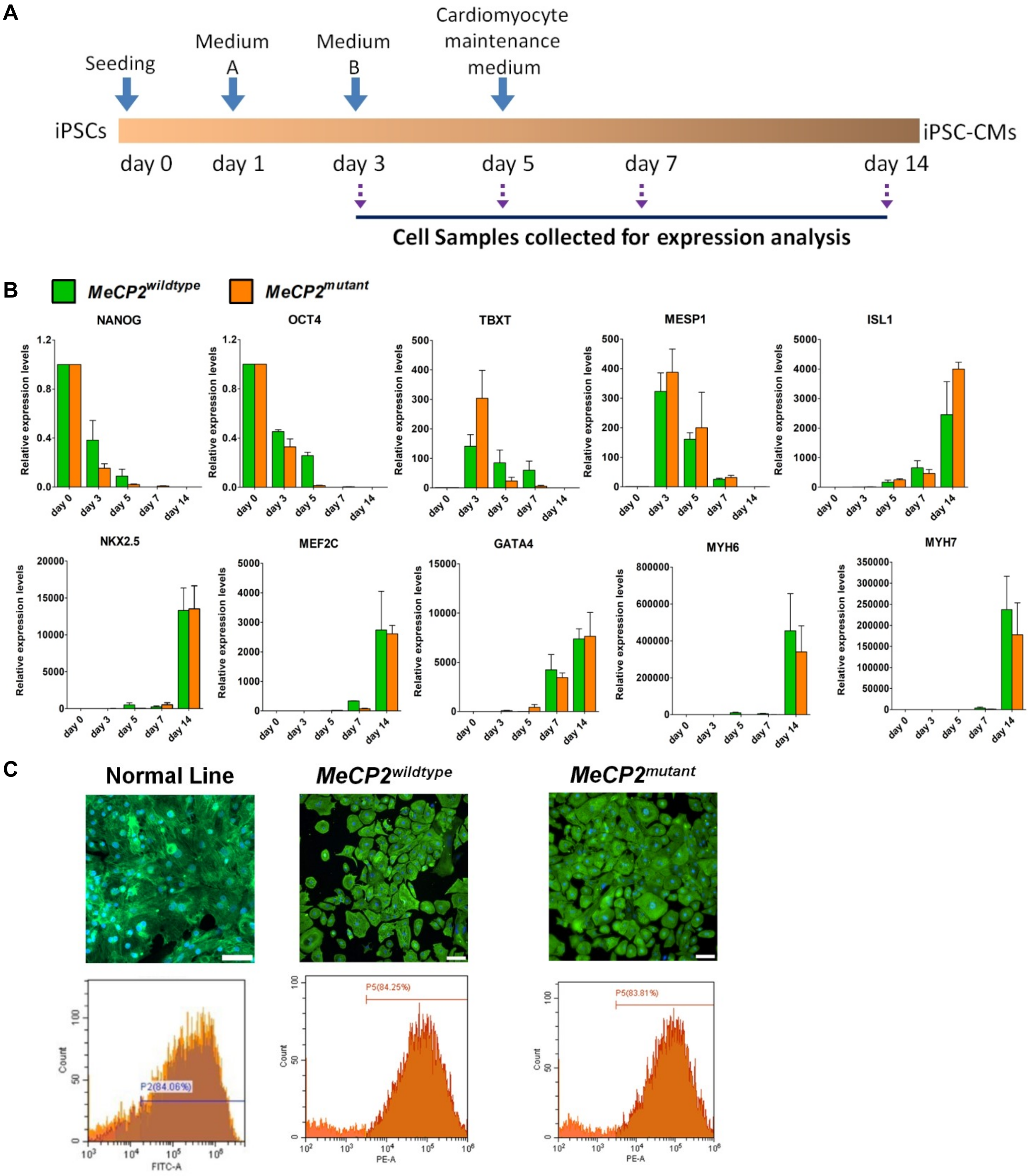

Figure S1. Differentiation of iPSCs into iPSC-CMs. (A) Outline of the cardiac differentiae protocol using the PSC cardiomyocyte differentiation kit (ThermoFisher). (B) Expressions of pluripotency (NANOG and OCT4), mesodermal (TBXT and MESP1) and cardiac markers (ISL1,NKX2.5,MEF2C,GATA4,MYH6 and MYH7) during cardiac differentiation by quantitative PCR analysis. (C) Evaluation of the percentages of cardiac-troponin T-positive cells by immunostaining and flow cytometry analysis using antibodies specific to human cardiac troponin-T (ThermoFisher, MA5-12960)

Figure S2, related to Figure 2

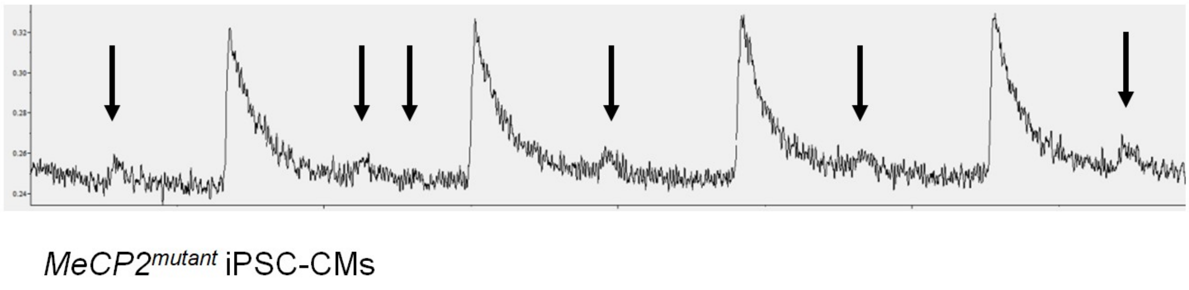

Figure S2. Tracing of calcium transient showing irregular peaks (indicated with arrow heads) in *MeCP2*^{mutant} iPSC-CMS.

Figure S3, related to Figure 3

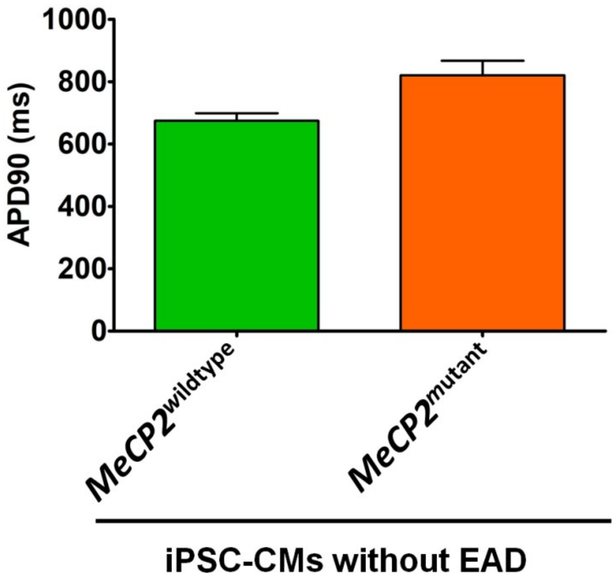

Figure S3. Action potential durations of the iPSC-CMs without EAD

Figure S4, related to Figure 6

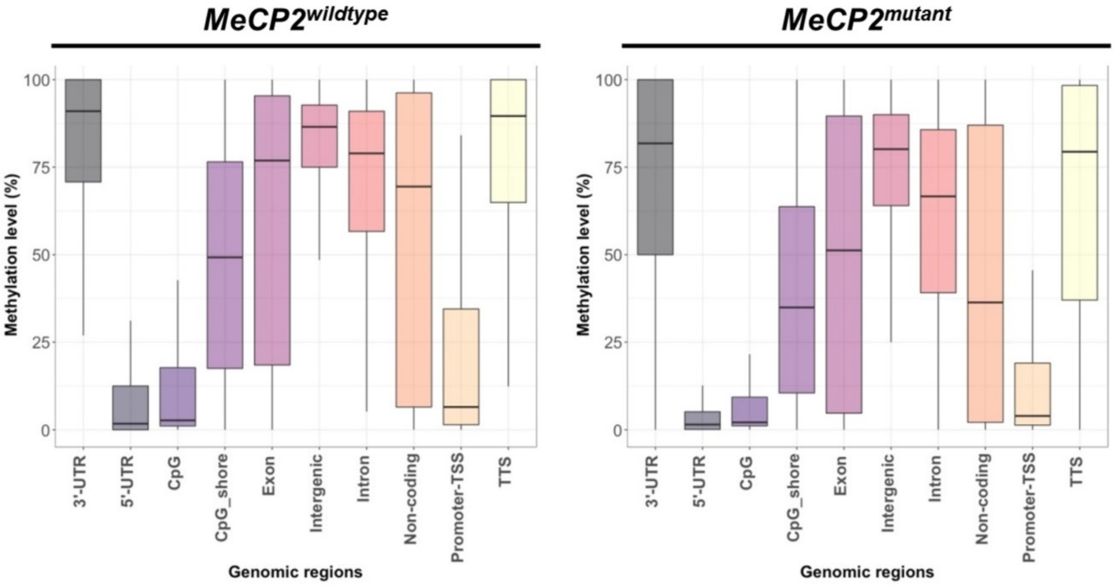

Figure S4. Methylation of different genomic regions in *MeCP2*^{wildtype} and *MeCP2*^{mutant}iPSC-CMs

Figure S5, related to Figure 7

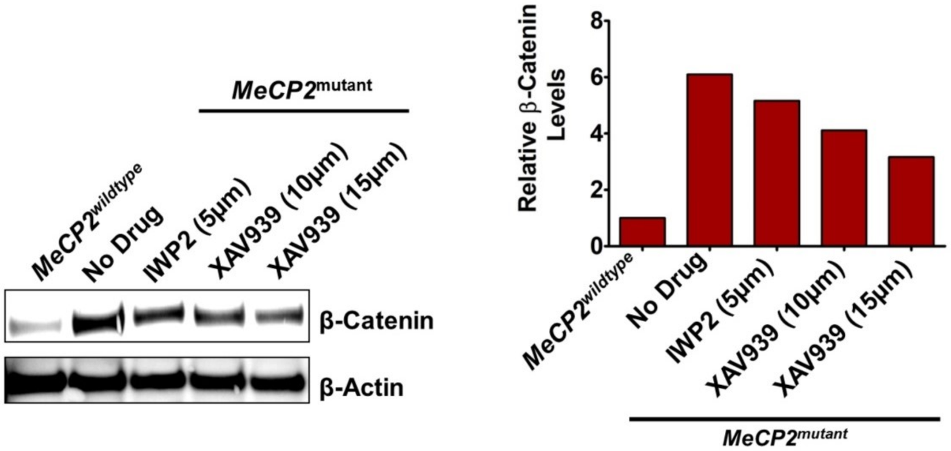

Figure S5. Evaluation of the effects of different WNT signaling inhibitors on β-catenin protein level in *MeCP2*^{mutant} iPSC-CMs
